# Supplementary material for: Integrated Physiological, Transcriptomic, and Metabolomic Analyses Revealed Molecular Mechanism for Salt Resistance in Soybean Roots
Source: Int J Mol Sci. 2021 Nov 27;22(23):12848. doi: 10.3390/ijms222312848 (PMC8657671; doi:10.3390/ijms222312848)
Supplement: Supplementary file 1 [file ijms-22-12848-s001.zip › ijms-1473606-supplementary.pdf]

Supplementary data

Table S1. The statistics of transcriptome sequencing data and map ratio.

Sample	Clean Reads	Mapped Reads	Uniq Mapped Reads	Multiple Map reads	Clean bases
C0JD-1	23,059,723	42,437,278 (92.02%)	41,220,268 (89.38%)	1,217,010 (2.64%)	6,900,047,292
C0JD-2	29,762,571	53,913,488 (90.57%)	52,570,040 (88.32%)	1,343,448 (2.26%)	8,904,906,492
C0JD-3	29,758,241	54,052,480 (90.82%)	52,513,254 (88.23%)	1,539,226 (2.59%)	8,902,685,772
C0LH-1	30,105,094	53,833,865 (89.41%)	52,216,300 (86.72%)	1,617,565 (2.69%)	9,002,714,328
C0LH-2	27,079,377	49,394,179 (91.21%)	47,978,977 (88.59%)	1,415,202 (2.61%)	8,102,619,962
C0LH-3	27,975,868	51,645,554 (92.30%)	50,219,087 (89.75%)	1,426,467 (2.55%)	8,372,295,860
C0LD-1	23,991,736	44,039,792 (91.78%)	43,005,232 (89.63%)	1,034,560 (2.16%)	7,177,897,948
C0LD-2	23,733,374	43,840,096 (92.36%)	42,411,148 (89.35%)	1,428,948 (3.01%)	7,105,281,174
C0LD-3	32,292,715	59,699,512 (92.43%)	58,230,948 (90.16%)	1,468,564 (2.27%)	9,656,362,532
S100JD-1	21,816,940	39,904,053 (91.45%)	38,852,630 (89.04%)	1,051,423 (2.41%)	6,525,631,586
S100JD-2	20,492,135	37,407,020 (91.27%)	36,557,977 (89.20)	849,043 (2.07%)	6,138,767,086
S100JD-3	23,890,450	44,311,149 (92.74%)	43,307,660 (90.64%)	1,003,489 (2.10%)	7,151,401,002
S100LH-1	24,891,135	45,972,845 (92.35%)	44,931,420 (90.26%)	1,041,425 (2.09%)	7,449,052,704
S100LH-2	24,189,726	44,617,029 (92.22%)	43,569,863 (90.06%)	1,047,166 (2.16%)	7,230,552,930
S100LH-3	24,545,861	45,334,351 (92.35%)	44,226,435 (90.09%)	1,107,916 (2.26%)	7,352,725,336
S100LD-1	22,248,727	41,720,437 (93.76%)	40,694,034 (91.45%)	1,026,403 (2.31%)	6,668,285,054
S100LD-2	21,687,505	40,214,480 (92.71%)	39,312,609 (90.63%)	901,871 (2.08%)	6,502,210,234
S100LD-3	21,683,915	40,655,787 (93.75%)	39,725,622 (91.60%)	930,165 (2.14%)	6,500,582,482

Note: (1) Sample analysis number, C0JD1, C0JD2, C0JD3 represent the three biological replicates of JD19 roots under the control condition, S100JD1, S100JD2, S100JD3 represent the three biological replicates of JD19 roots under the salt stress conditions, LH3 and LD2 are the same as JD19; (2) Clean reads represent the total number of pair end reads in clean data; (3) Mapped Reads represent the number of reads compared to the reference genome and its percentage in clean reads; (4) Uniq Mapped Reads represent the number and

percentage of clean reads compared to the unique position of reference genome; (5) Multiple Map Reads represent the number and percentage of clean reads compared to the multiple position of reference genome; (6) Clean bases represent the total base of clean data.

Table S2. qRT-PCR confirmation of RNA-seq data.

Relative expression of representative genes obtained using qRT-PCR of three independent biological replicates.						
gene_name	JD0 vs JD100		LH0 vs LH100		LD0 vs LD100	
	FC	SD	FC	SD	FC	SD
CAT5	2.52378127	0.256593073	2.030811985	0.19578979	1.4058755	0.043056577
SAT1	2.469148552	0.092933332	2.034574276	0.058955541	1.648273124	0.099812093
LOC100786514	2.096185628	0.182673585	1.96154616	0.089576744	1.515716567	0.073543027
LOC100775844	3.527171322	0.55955038	2.965936329	0.013390721	1.949046407	0.022834198
LOC100170730	1.932588923	0.077729928	1.820741609	0.017066842	1.830428634	0.038565821

Fold changes in expression of genes were obtained from transcriptome analysis.						
gene_name	JD0 vs JD100		LH0 vs LH100		LD0 vs LD100	
	DESeq_FDR	log_2FC	DESeq_FDR	log_2FC	DESeq_FDR	log_2FC
CAT5	1.15339E-05	1.317568909	8.49407E-09	1.043397351	0.108602988	0.634505227
SAT1	3.58425E-08	1.363929603	1.74496E-07	1.039994001	0.005427297	0.78946753
LOC100786514	0.000584549	1.000220003	1.74348E-05	1.011085681	0.067076762	0.725545969
LOC100775844	8.06467E-07	1.826212926	3.72637E-06	1.575739228	0.083334079	1.282336328
LOC100170730	0.006413062	1.137937704	0.00011143	1.27872378	0.121407732	1.000134272

Table S3. Relative concentrations and fold-changes in the levels of differentially expressed metabolites in roots of JD19, LH3 and LD2 under salt stress. Fold changes were calculated using the formula log2(100/0). *, **; *** means significant differences at P<0.05; P<0.01 and P<0.001, respectively.

Metabolites name	JD19		
	Relative concentration		Fold Changes
	0 mM NaCl	100 mM NaCl	Log2 (100/0)
fructose	86884.98±15443.82	39564.58±6633.17	-1.13*
proline	23129.18±2184.69	44215.09±3257.73	0.93***
oxoproline	28819.11±5916.58	48706.75±793.65	0.76*
citric acid	8073.88±622.81	17130.99±1181.15	1.09***
malonic acid	10233.06±1892.93	20187.26±3336.20	0.98*
mannose	30078.29±3610.02	19021.26±3061.33	-0.66*
L-Malic acid	10691.97±784.81	5159.29±370.54	-1.05***
Aspartate	17610.14±706.99	22979.67±743.69	0.38***
beta-Alanine	5308.25±441.21	8317.20±791.51	0.65**
Saccharic acid	3913.71±843.76	6531.57±105.23	0.74*
L-Allothreonine	12061.48±407.62	10150.78±472.99	-0.25*
4-aminobutyric acid	3497.12±457.00	5389.76±436.14	0.62*
Threonine	1571.91±145.43	2603.07±167.86	0.73**
L-homoserine	1798.26±195.07	767.73±185.18	-1.23**
	LH3		
	Relative concentration		Fold Changes
	0 mM NaCl	100 mM NaCl	Log2 (100/0)
fructose	82932.95±6823.84	41950.81±9009.35	-0.98**
sorbose	106739.70±8576.40	67640.33±6731.53	-0.66**
oxoproline	39966.14±3005.19	52734.24±3951.61	0.40*

proline	20816.19±2667.28	33023.73±3695.82	0.67*
malonic acid	7813.64±724.37	16530.68±1739.45	1.08***
mannose	34270.97±1945.77	26698.05±2326.20	-0.36*
citric acid	5856.38±529.15	12255.37±894.80	1.07***
beta-Alanine	5703.72±997.08	11181.40±558.25	0.97***
Tagatose	19933.24±1992.47	14689.54±1029.90	-0.44*
Aspartate	24048.72±2490.38	17826.35±1069.61	-0.43*
phenylalanine	5900.47±777.24	9665.41±774.60	0.71**
L-Malic acid	7457.89±439.74	4400.26±503.06	-0.76**
4-aminobutyric acid	3629.67±472.95	6106.28±1005.86	0.75*
Threonine	1329.02±289.89	2780.41±292.28	1.06**

	LD2		
	Relative concentration		Fold Changes
	0 mM NaCl	100 mM NaCl	Log2 (100/0)
proline	17183.88±1540.49	28154.82±936.56	0.71***
Aspartate	29359.89±1384.00	20510.27±897.96	-0.52***
L-Malic acid	11732.16±913.86	4117.90±269.13	-1.51***
valine	42217.09±1851.37	34665.69±2656.21	-0.28*
citric acid	6843.08±323.89	13036.98±1079.09	0.93***
succinic acid	14845.46±1335.53	9880.75±1075.50	-0.59*
tryptophan	6565.61±721.11	11378.46±1126.38	0.79**
Ethanolamine	19982.76±1148.83	16490.81±713.42	-0.28*
palmitic acid	16872.85±560.27	12445.69±1521.83	-0.44*
2-hydroxypyridine	9664.27±869.58	7307.60±521.67	-0.40*
Threonine	2278.65±125.92	4332.02±458.95	0.93**
stearic acid	8421.76±427.99	6678.00±488.38	-0.33*
2-ketobutyric acid	4786.06±212.44	3681.50±257.38	-0.38**

L-homoserine	1148.78±96.15	515.57±130.05	-1.16**

Table S4. Characteristics of the three soybean cultivars.

Cultivars	Location	Year of release	Characteristics
JINDOU19 (JD19)	Shanxi	2003	Few branches, large seed size
LONGHUANG3 (LH3)	Gansu	2018	Few branches, large seed size
LONGDOU 2 (LD2)	Gansu	2005	Several branches, small seed size

Table S5. Primer sequences used in the study

Gene name	Primer sequences
GmCAT5	Forward: 5'-CGGGTCACCCATGAAATCCG-3'<br>Reverse: 5'-ATTTGGCCTCATGCTCAGAC-3'
GmSAT1	Forward: 5'-GACATCGTAGGTGCTGTGAAG-3'<br>Reverse: 5'-CCTCCCTTGAAGCCACAATTT-3'
LOC100786514	Forward: 5'-TTAGTGGCCTATCGAGCACC-3'<br>Reverse: 5'-AACTACGCATCACAGCAGCA-3'
LOC100775844	Forward: 5'-AACCAATCTCCAAACCCGTG-3'<br>Reverse: 5'-GACTTGGCACGGAATGCG-3'
LOC100170730	Forward: 5'-TCAGCCCTTTGCTGTTTTCACT-3'<br>Reverse: 5'-GCTTGTTGATGCTACCCTTCTTC-3'
GmACTIN2	Forward: 5'-AAGGGTATTGCTGCTGGCTT-3'<br>Reverse: 5'-CAGGGGAGGGAAAAACACCC-3'

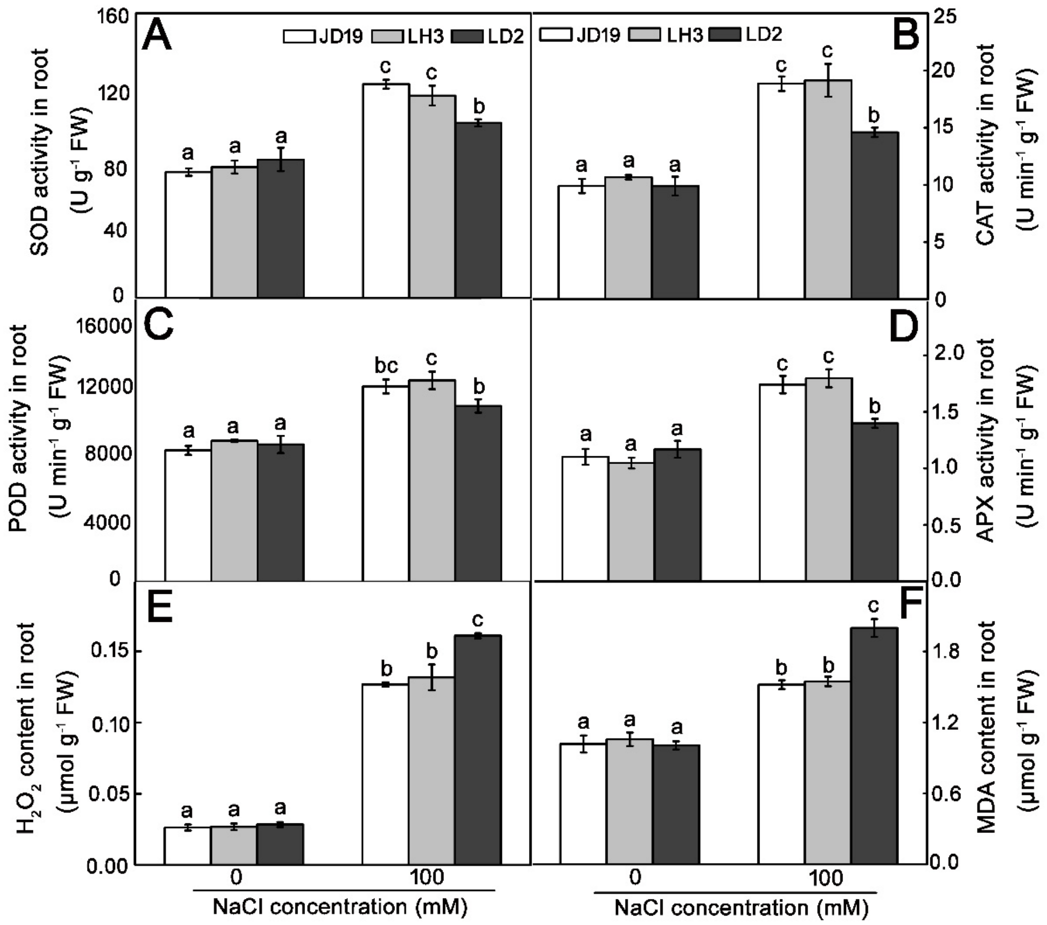

Figure S1. Activities of SOD (A), CAT (B), POD (C) and APX (D), contents of H_2O_2 (E) and MDA (F) in roots of three soybean cultivars under 100 mM NaCl treatment. Data are means ± SE (n=3, P<0.05). Different letters indicate significant difference at $p < 0.05$.

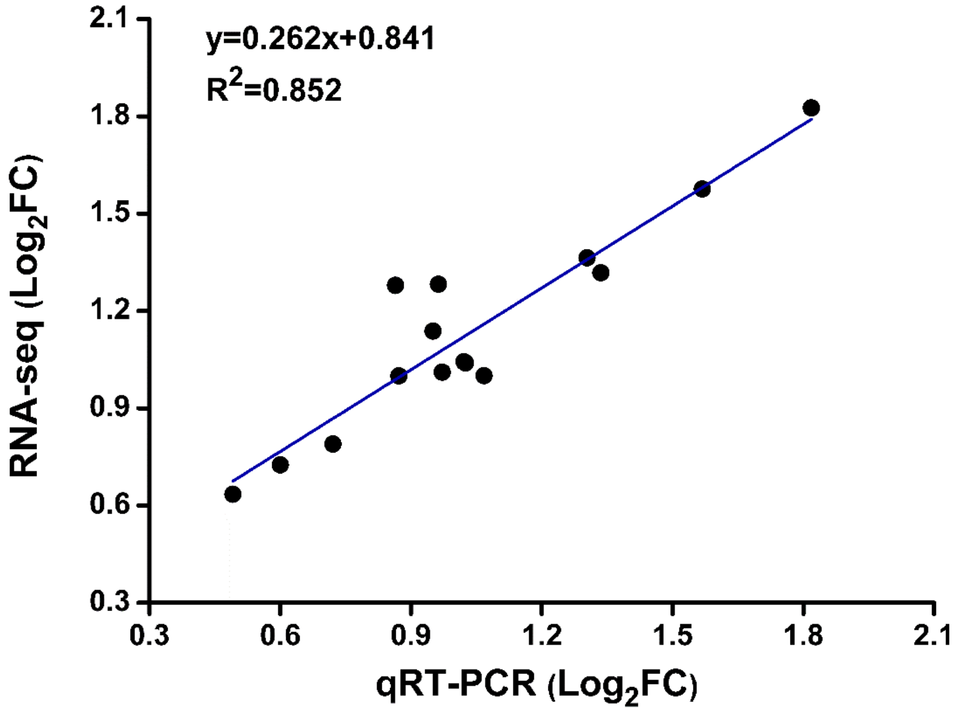

Figure S2. Correlation analysis between qRT-PCR and RNA-seq data.

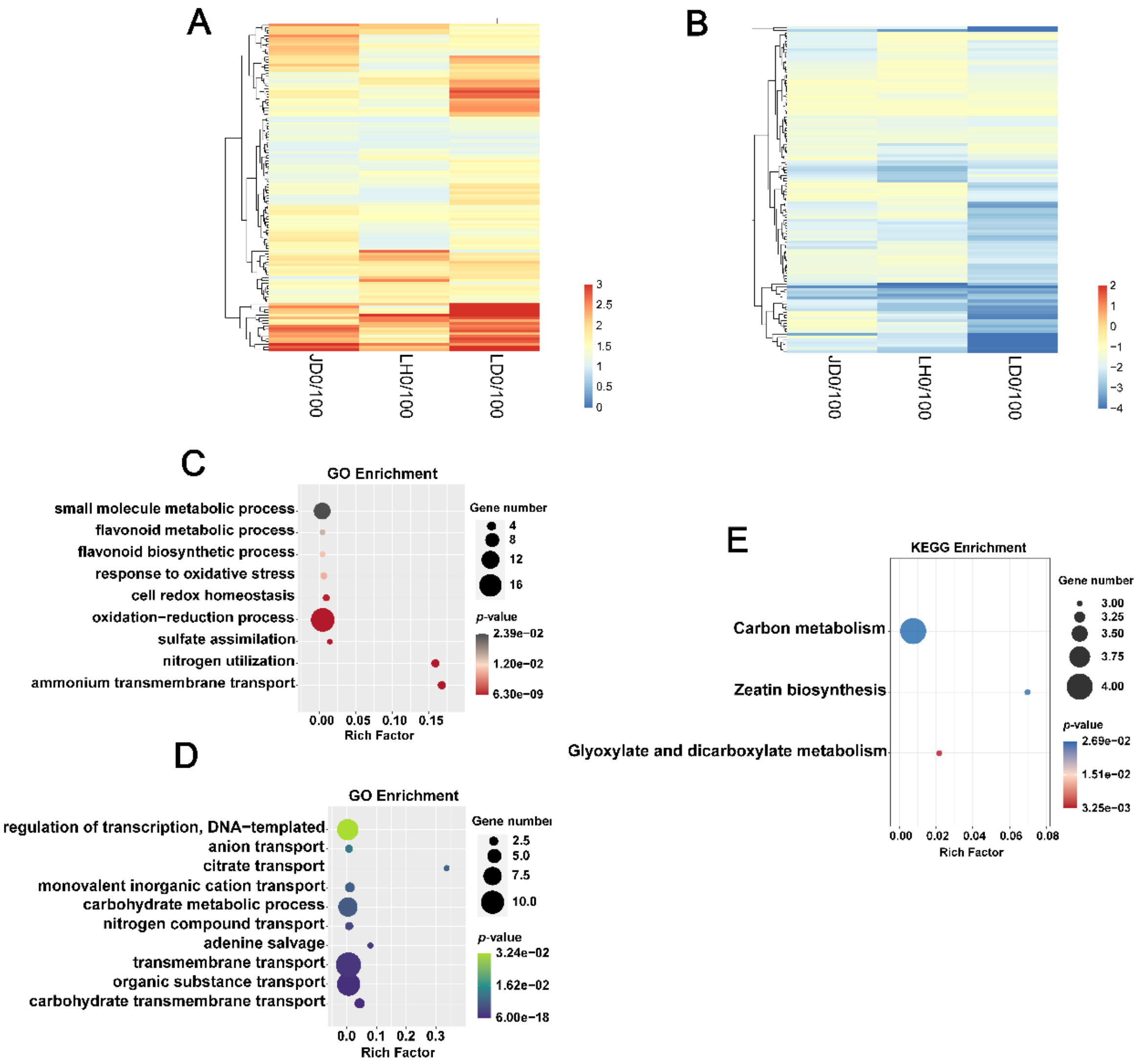

Figure S3. Analysis of common regulated DEGs in three soybean cultivars under salt stress. Heatmap of common up-regulated DEGs (A), common down-regulated DEGs (B), GO enrichment of common up-regulated DEGs (C), common down-regulated DEGs (D), KEGG enrichment of common up-regulated DEGs (E) among three soybean cultivars under salt stress.

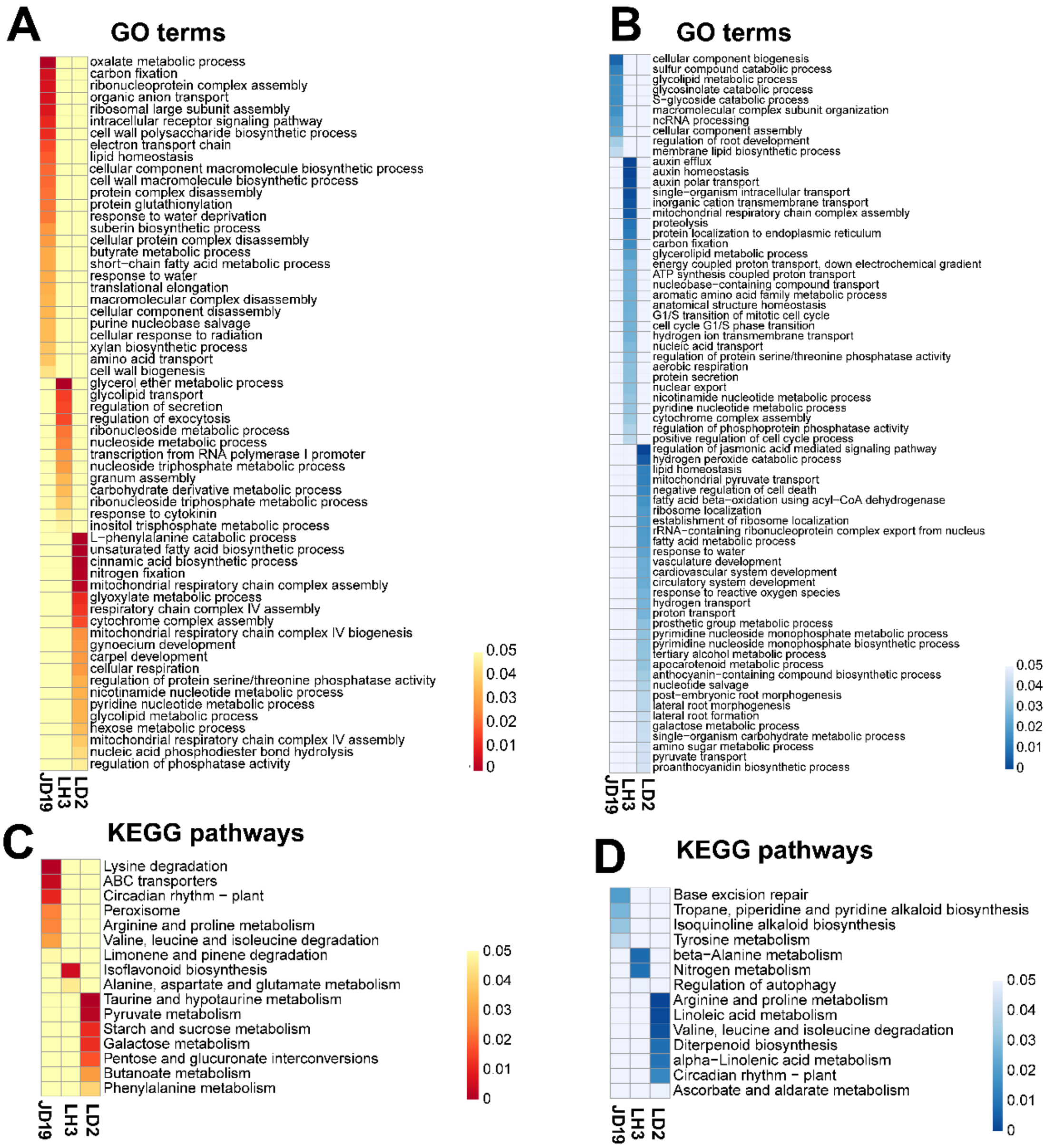

Figure S4. Analysis of independent regulated DEGs in three soybean cultivars under salt stress. GO term of independent up-regulated DEGs (A), independent down-regulated DEGs (B), KEGG pathway of independent up-regulated DEGs (C), independent down-regulated DEGs (D) among three soybean cultivars under salt stress.
